# Incidentally discovered intestinal malrotation during evaluation for blunt abdominal trauma: A case report

**DOI:** 10.1016/j.ijscr.2024.109430

**Published:** 2024-02-22

**Authors:** Shishir Devkota, Prajjwol Luitel, Sujan Paudel, Nischal Neupane, Santosh Dev, Prasan Bir Singh Kansakar

**Affiliations:** a Department of General Surgery, Maharajgunj Medical Campus, Tribhuvan University Teaching Hospital, Nepal; b Maharajgunj Medical Campus, Institute of Medicine, Tribhuvan University, Kathmandu, Nepal

**Keywords:** Embryology, Incidental finding, Malrotation, Trauma

## Abstract

**Introduction and importance:**

Intestinal malrotation is a congenital abnormality predominantly diagnosed in children, with only a few cases reported in adults. Patients may be incidentally identified during unrelated surgical procedures or postmortem examinations. It is crucial to promptly recognize this condition to prevent severe complications such as bowel ischemia and potential fatality.

**Case presentation:**

A 40-year-old male presented to the Emergency Department after a child jumped on his abdomen with complaints of acute left upper quadrant abdominal pain progressing to be generalized. Examination showed pallor, abdominal tenderness without guarding or rigidity, and intact bowel sounds. Preoperative diagnostic tools revealed intestinal malrotation confirmed during the laparotomy, prompting the performance of Ladd's procedure to address the malrotation.

**Clinical discussion:**

Disruption in the normal embryological development of bowel is the cause of intestinal malrotation. The role of additional surgery especially in patients with asymptomatic disease related to malrotation is debated.

**Conclusion:**

Intestinal malrotation is rare in adults and often found incidentally during evaluation for unrelated medical conditions. Timely identification and surgical intervention usually result in positive outcomes. Our case underscores the incidental discovery of malrotation during the evaluation of blunt abdominal trauma, treated with Ladd's procedure. This is particularly significant due to geographical constraints associated with the patient's rural origin, as untreated malrotation could lead to complications in future occurrences.

## Highlights

• Adult intestinal malrotation is rare with the majority of patients remaining asymptomatic.
• Surgeons need a comprehensive understanding of embryology, especially gastrointestinal and vascular anatomy for malrotation.
• We present the discovery of malrotation during evaluation for blunt abdominal trauma managed with Ladd’s procedure.

## 1 Introduction

Intestinal malrotation is characterized by a lack of rotation or incomplete rotation around the superior mesenteric artery, coupled with abnormalities in intestinal fixation [1]. While the estimated incidence of intestinal malrotation is 1 in 200 to 1 in 500 live births, symptomatic cases are observed in only about 1 in 6000 live births [2,3]. It is typically diagnosed in early life, with 80 % of cases presenting in the first month and 90 % within the first year the occurrence of malrotation in adults is infrequent, affecting approximately 0.2 % of the population [4,5].

It manifests with symptoms like acute bowel obstruction, intestinal ischemia, chronic abdominal pain, duodenal obstruction, and internal herniation [6]. Yet, diagnosis becomes challenging in asymptomatic patients, and various modalities, such as barium studies, CT scans, angiography, and sometimes emergent laparotomy, are employed for identification. Conversely, many adults with malrotation remain undiagnosed throughout their lives, as they exhibit no symptoms. These cases are often incidentally identified during unrelated surgical procedures or postmortem examinations [7].

Blunt traumatic incidents, such as motor vehicle collisions, motorcycle crashes, falls, and assaults, often result in polytrauma and extensive soft tissue injuries [8]. Trauma patients undergo various assessments, including X-rays, ultrasound, CT scans, or laparotomy, to detect hidden injuries. Incidental findings may emerge during these diagnostic procedures. This paper, following SCARE guidelines, details a unique case where congenital intestinal midgut malrotation was incidentally discovered during evaluation for hemoperitoneum in an adult patient following blunt force trauma [9].

## 2 Case presentation

A 40-year-old male, with no significant medical or surgical history, presented to the Emergency Department after an incident involving a child (weighing 12 kg) jumping onto his abdomen. Subsequently, the patient experienced acute onset left upper quadrant abdominal pain that was continuous, non-radiating, aggravated on inspiration, and relieved with analgesics. Over the course of a day, the pain progressed to generalized abdominal pain. There were no reported symptoms of abdominal distention, nausea, vomiting, loss of consciousness, or fever at the time of presentation. Vital signs upon arrival included a pulse of 122 bpm, blood pressure of 90/50 mmHg, respiratory rate of 18 per minute, temperature of 98 °F, and oxygen saturation of 97 % in room air. The patient appeared pale with abdominal tenderness over all quadrants without guarding or rigidity. Bowel sounds were present.

Initial blood investigations revealed a platelet count of 95,000/mm^3^ and a hemoglobin level of 6 g/dl. Other blood investigation parameters were within normal limits at the initial presentation. An Extended Focused Assessment with Sonography for Trauma (*E*-FAST) was performed, revealing hemoperitoneum.

**Fig. 1:**
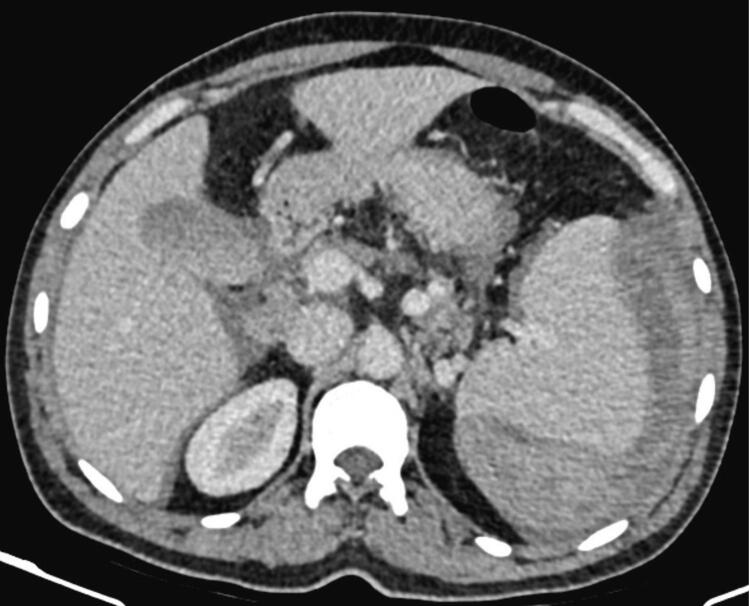
Contrast-enhanced computed tomography (CECT) of the abdomen, arterial phase (axial view) revealing splenic contrast blush.

**Fig. 2:**
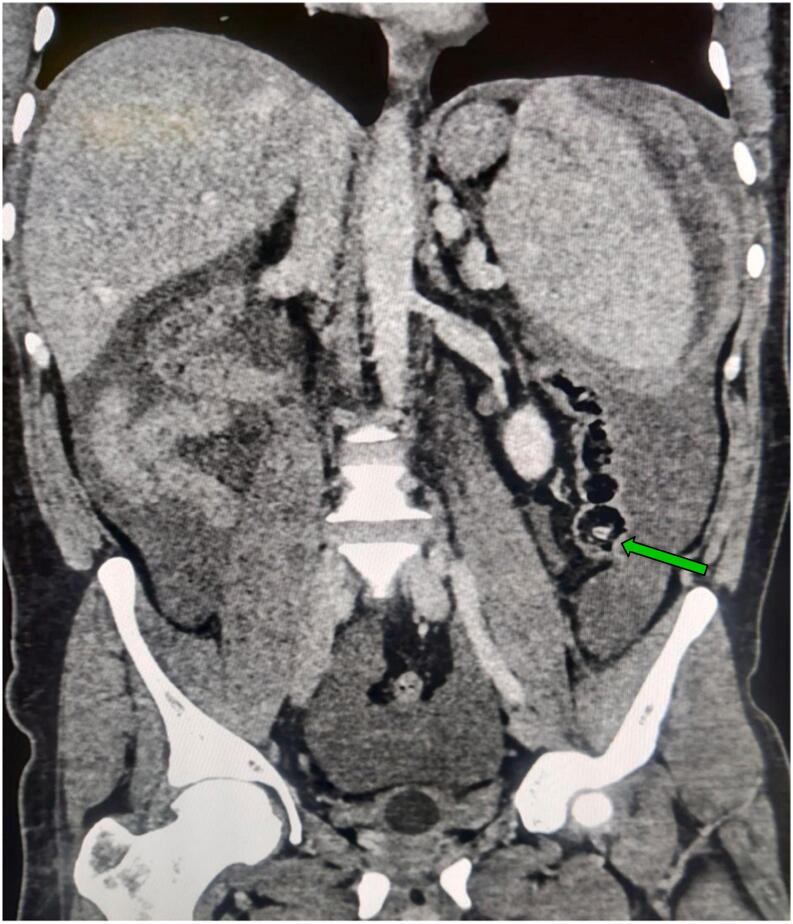
Contrast-enhanced computed tomography (CECT) of the abdomen and pelvis, venous phase, coronal section showing cecum in the left lower quadrant.

**Fig. 3:**
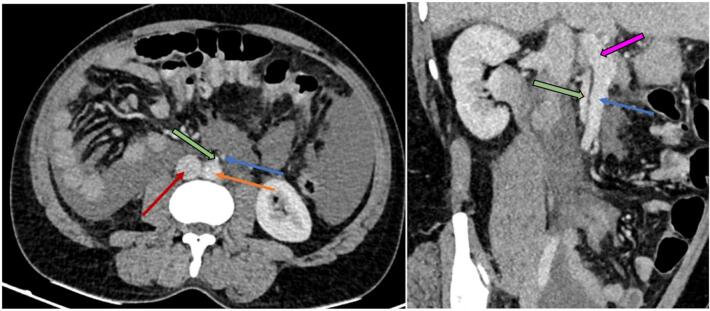
Contrast-enhanced computed tomography (CECT) of the abdomen and pelvis, venous phase, axial (left) and oblique coronal (right) sections. Inferior vena cava (IVC) is labeled in red, the aorta in orange, the superior mesenteric artery (SMA) arising from the aorta in green, and the superior mesenteric vein (SMV) in blue, portal vein in pink; demonstrating SMA is right to SMV typical for intestinal malrotation. (For interpretation of the references to colour in this figure legend, the reader is referred to the web version of this article.)

The patient was initially resuscitated with 2 units of intravenous fluids and received 2 units of packed red blood cells resulting in an increase in hemoglobin to 7 g/dl. Additionally, the patient was administered intravenous tranexamic acid, analgesics, antiemetics and antibiotics. Following initial resuscitation, a contrast enhanced computed tomography (CECT) of the abdomen was done, revealing a splenic intraparenchymal injury with contrast extravasation (Fig. 1). In addition, the CECT exhibited the cecum situated within the left lower quadrant of the abdomen (Fig. 2), and a reversal of the typical arrangement between the superior mesenteric artery (SMA) and the superior mesenteric vein (SMV), suggestive of intestinal malrotation (Fig. 3).

**Fig. 4:**
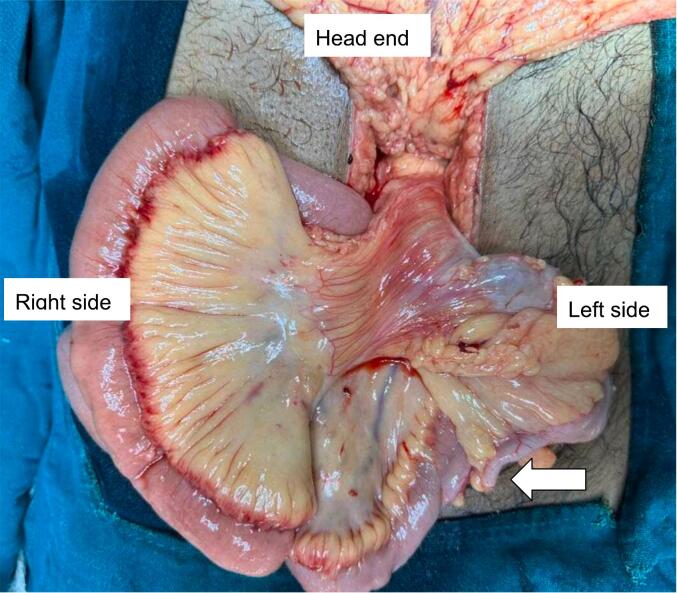
Intraoperative image depicting the presence of the cecum on the left side, along with the appendix (indicated by the arrow).

**Fig. 5:**
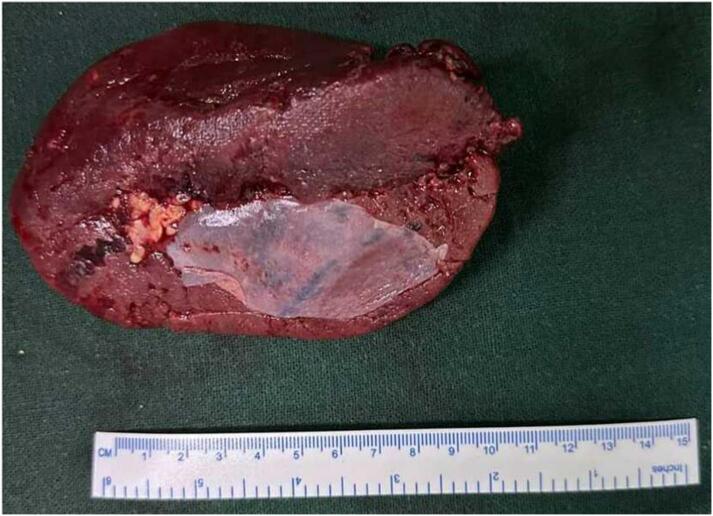
Surgical specimen following splenectomy.

An emergency exploratory laparotomy was performed. Intraoperatively, an unusual arrangement of the SMA and SMV was observed, with the vein positioned to the left of the artery and the cecum located in the left lower quadrant, indicating the likelihood of malrotation (Fig. 4). Ladd's procedure was performed along with an appendectomy and splenectomy (Fig. 5). The postoperative course was uneventful and the patient was discharged on the tenth postoperative day. During a follow-up appointment at two weeks, the patient received the pneumococcal vaccine (PCV20), the *Haemophilus influenzae* type b vaccine (Hib), and the meningococcal vaccine (MenACWY). Throughout the 12-month follow-up period, the patient remained devoid of abdominal complaints.

## 3 Discussion

Intestinal malrotation results from a failure of rotation and fixation of bowel segments during normal embryological bowel development, leading to abnormalities in the positioning and attachment of the intestines [10]. The most common form is nonrotation, where the caecal bud is the initial segment returning into the abdomen, ultimately settling in the left lower quadrant. Acute symptoms include intense abdominal pain caused by volvulus. In contrast, chronic malrotation symptoms often manifest as vague intermittent abdominal pain, nausea, vomiting, dyspepsia, gradual weight loss, and unexplained abdominal discomfort. Unfortunately, chronic malrotation symptoms are frequently misdiagnosed, with as many as 83 % of adult patients remaining asymptomatic [11]. Discovery of the anomaly commonly occurs incidentally during autopsy or surgery.

In adult populations, computed tomography (CT) scans, particularly when administered with intravenous and oral contrast, offer superior diagnostic utility, with certain research advocating for CT as the primary imaging modality in suspected cases of malrotation. CT not only reveals abnormal indicators that may be visible on upper gastrointestinal and ultrasound scans but also circumvents the impact of intestinal gas, allowing for comprehensive anatomical assessment of the bowel [12,13]. In our case, CECT identified rotational midgut anomaly. While intestinal malrotation, when present, may coincide with other anatomical abnormalities, such as a wandering liver, central nervous system abnormalities, genitourinary malformations, or recurrent pancreatitis increasing clinical suspicion, our case did not exhibit such abnormalities [14].

Surgery is widely recognized as the appropriate intervention for addressing symptomatic conditions directly associated with malrotation. However, ongoing debate exists about the role of surgery, particularly for patients with asymptomatic disease [15]. Choosing to forgo surgical treatment may result in a lifetime risk of emergency surgery for acute volvulus or ischemia, estimated to be as high as 20 %. Much of the current surgical literature advocates for operative correction in cases with no or minor symptoms or incidental discoveries [16,17]. The preferred treatment involves surgically resecting the affected bowel segment and performing Ladd's procedure. This procedure includes lysis of adhesive bands to mobilize the midgut, counterclockwise reduction of the volvulus, widening of the midgut mesenteric pedicle, and appendectomy [18]. A review of the English literature identified a single previous instance of congenital intestinal malrotation detection post-blunt abdominal trauma through radiological means [8]. However, in that case, the patient was discharged after 24-h observation and remained asymptomatic afterward. In contrast, our case exhibited active contrast blush on contrast-enhanced computed tomography (CECT), prompting an emergency laparotomy and splenectomy to address the splenic injury. Additionally, we performed a Ladd's procedure due to geographic constraints associated with the patient's rural origin, as leaving the condition untreated could potentially lead to complications in subsequent occurrences.

## 4 Conclusion

Intestinal malrotation in adult patients is a rare finding, often identified incidentally during radiological examinations or surgical exploration for another disease process. Although its diagnosis poses challenges, timely identification and surgical intervention often lead to favorable outcomes. Currently, there is no established standard for the surgical management of intestinal malrotation in adults. Our case offers a unique contribution to the literature by showcasing the discovery of malrotation during evaluation for blunt abdominal trauma and the subsequent successful management thereof.

## Consent

Written informed consent was obtained from the patient for publication and any accompanying images. A copy of the written consent is available for review by the Editor-in-Chief of this journal on request.

## Ethical approval

Since this is a case report, our Institutional Review Board has waived the requirement for ethical approval.

## Funding

No funding received.

## Author contribution

Conceptualization: Dr Santosh Dev, Dr Shishir Devkota.

Patient Management: Dr Santosh Dev, Dr Shishir Devkota, Dr Prasan Bir Singh Kansakar

Writing – original draft: Dr Santosh Dev, Dr Shishir Devkota, Dr Sujan Paudel, Dr Prajjwol Luitel, Dr Nischal Neupane

Writing – review & editing: Dr Santosh Dev, Dr Shishir Devkota, Dr Sujan Paudel, Dr Prajjwol Luitel, Dr Barsha Dev, Dr Nischal Neupane

Visualization and Supervision: Dr Santosh Dev, Dr Shishir Devkota, Dr Prasan Bir Singh Kansakar

## Guarantor

Prajjwol Luitel.

## Conflict of interest statement

The authors declare that there is no conflict of interest regarding the publication of this article.
